# Professionalism and associated factors among nurses working in Hawassa city public hospital, Sidama, Ethiopia

**DOI:** 10.3389/fmed.2024.1352499

**Published:** 2024-08-22

**Authors:** Eyerusalem Abebe Boe, Shiwangizaw Mekonnen, Thomas Fako, Mastewal Aschale Wale, Meku Tade, Aklile Tsega Chekol

**Affiliations:** ^1^Faculty of Health Sciences, College of Medicine and Health Sciences, Hawassa University, Hawassa, Ethiopia; ^2^Department of Anesthesia, College of Medicine and Health Sciences, Hawassa University, Hawassa, Ethiopia

**Keywords:** professionalism, associated factors, public hospitals, Sidama, Ethiopia

## Abstract

**Background:**

The foundation of the global healthcare system is nurses, and professionalism in nursing is a basic idea that helps patients, organizations, and people. Studies that have been published in Ethiopia, though, are limited, out-of-date, and poorly documented, especially when it comes to the study setting. Because of this, this study aimed to close a knowledge gap on the level of professionalism in public hospitals in Sidama, Ethiopia.

**Objective:**

This study aimed to assess professionalism and associated factors among nurses working in Hawassa city public hospitals, Hawassa, Ethiopia.

**Methods:**

An institutional-based cross-sectional study was conducted among nurses working in Hawassa city public hospital from June to July 2022. A computer-generated simple random sampling technique was used to select 413 study participants. The level of professionalism was assessed through a self-administered questionnaire, using the guidelines of the Registered Nurses Association of Ontario. All the loaded data using Epi-data version 4.6 were exported to a statistical package for social science. An ordinal logistic regression analysis was used to identify the associations between the outcome and predictor variables. The statistical significance of the factors influencing the outcome variable was declared in multivariate logistic regression analysis using an adjusted odds ratio at a 95% confidence interval with a *p*-value <0.05.

**Results:**

A total of 405 nurses participated in the study, with a response rate of 98%. Of the total participants, more than half were females (55.3%). The level of professionalism was found to a moderate level. There was a strong link between completing their degree in a governmental institution, being part of a professional organization, serving for several years, and having a BSc or above qualification with a moderate level of professionalism.

**Conclusion:**

We found a moderate level of professionalism among nurses working in the study setting. This suggests that the Regional Health Bureau should collaborate with other responsible bodies to develop various opportunities for nursing staff to increase their professionalism. The minister of health should be focused on private college nurses, nurses lacking the association, and the qualification of the profession.

## Introduction

The foundation of the global healthcare system is nurses, and professionalism in nursing is a fundamental idea that helps patients, organizations, and people (1, 2). In the healthcare sector, nurses are on the front lines of care at all levels (1). Professionalism is understood as the behavior, objectives, or characteristics that characterize a profession or a professional individual (3). It is a conceptualization of the responsibilities, traits, relationships, perceptions, autonomy, creative thinking, qualities, interactions, attitudes, and positional behavioral patterns required of professionals in their interactions with people and society at large (4–7).

Professionalism in nursing is crucial for achieving the main objectives of any healthcare delivery setting, such as providing high-quality care, raising patient satisfaction, enhancing the public’s perception of the industry, and meeting health-related indicators, as well as fostering efficient teamwork, the best possible patient outcomes, and job satisfaction (8). It is about showing commitment and willingness to continuously deliver the high-quality care for patients (9). It includes a wide range of personal characteristics such as self-regulation and professional values, as well as the pursuit of expert knowledge, professional interactions, social, professional, and legal responsibility, and the development of a sense of belonging and professional development (10).

Professionalism in nursing has been found to enhance patient happiness and health outcomes, which enhances nurses’ performance and job satisfaction (11). On the other hand, a lack of professionalism has been connected to detrimental outcomes such as higher turnover and attrition as well as lower productivity because it can erode trust between professionals and clients, diminish professional reputability, and possibly even cause a loss of self-regulation (12).

Despite the focus on quality care in the modern world, nursing professionalism faces several obstacles, such as quick changes in nursing practice, membership, communication, population diversity, inadequate leadership abilities, a lack of self-determination, healthcare risks, lengthy working hours, sentimental load, lack of appreciation by society, a shortage of nurses, and limited job advancement (1). Due to the attributes attached to professionalism in nursing, a nurse’s level of professionalism can have a significant impact on the outcome of patient care and the proportion of the healthcare team (13).

The decline in nursing professionalism has a wide range of consequences, including a reduction in teamwork, quality patient care, communication, collaboration, and job satisfaction; an increase in incivility; and also negative outcomes, including increased turnover and attrition and decreased productivity (14). As a result of a low level of professionalism, the tendency to make medical errors has increased by 25% (15). The majority of mistakes made by nurses are the result of unprofessional actions, such as failing to provide the necessary care and services, carrying out their job inadequately or poorly, performing a service or procedure they are not required to perform, acting carelessly and lacking the necessary skills, and lacking in knowledge and experience. Serving people, their families, healthcare providers, institutions, and nations suffer greatly as a result of these mistakes (16, 17). In Ethiopia, the level of professionalism among nurses was reported as 24.8, 30.3, and 58.7% (2, 18, 19). Even though there are limited studies published in Ethiopia, those are outdated, and there are differences in the level of the nursing profession. Because of this, the goal of this study was to close a knowledge gap on the degree of nursing professionalism working in public hospitals in Hawassa city as well as the characteristics that are related to professionalism.

### Study design, period, and setting

An institutional-based cross-sectional study was employed from June to July 2022, in Hawassa city public hospitals. Hawassa is the capital city of the Sidama national regional state, which is far by 273 km from the capital city of the country, Addis Ababa. According to the Community Support Agriculture (CSA) (2007), the total population of the city is 371,826 residing in eight sub-cities. There are four public hospitals in Hawassa city (Hawassa University Comprehensive Specialized Hospital, Adare General Hospital, Tula Primary Hospital, and Motite Furra Primary Hospital). The total of nurses working in this hospital was 787. Of these, 494 were from Hawassa University Comprehensive Specialized Hospital, 180 were from Adare General Hospital, 73 were from Tula Primary Hospital, and 40 were from Motite Furra Primary Hospital.

### Study population

All nurses working in Hawassa city public hospitals were the source population, and all selected nurses who were on active duty during the study period were the study population.

### Inclusion and exclusion criteria

All selected nurses who were on active duty during the study period were included. On the other hand, nurses who were on annual or maternal leave were excluded.

### Sample size determination and sampling technique and procedure

The sample size for this study was calculated using the single population proportion formula by considering the following assumptions: The population proportion (*p*) was 57.8% (19) from the previous study, 95% CI (1.96), and 5% margin of error.

$$n=n\frac{\left(Za/2\right)2\;P\left(1-P\right)}{d2}:n=\frac{{\left(1.96\right)}^{2}\;0.578\left(0.422\right)\;}{{\left(0.05\right)}^{2}}:n=375$$

After adding a 10% non-response rate, the final sample size was 413.

**Figure 1:**
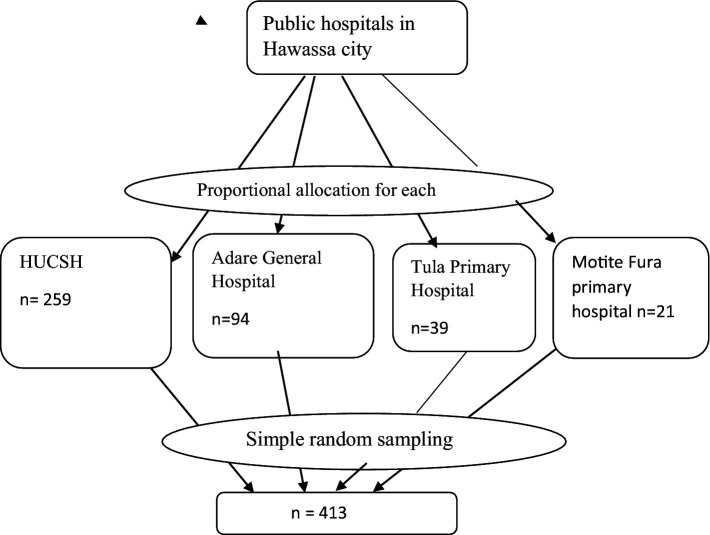
Proportional allocation of nurses working in Hawassa city public hospitals, in 2022 (*n* = 405).

All public hospitals in Hawassa city were included. The lists of nurses who were recently working in each hospital were taken from each hospital’s human resources and management unit. Finally, the respondents were selected using a simple random sampling technique. To select each study unit, a computer-generated simple random sampling technique was used (Figure 1).

### Operational definitions

“High level of professionalism” = score between 80 and 100%; “moderate level of professionalism” = score between 60 and 79%; and “low level of professionalism” = score less than 60% (18).

### Data collection tools

The data were collected using the English language through a semi-structured, self-administered questionnaire by four nurse professionals. The questionnaire had three parts: The first part consisted of the socio-demographic characteristics of the study participants. The second part consisted of 34 items on a self-appraisal scale to measure the outcome variable of professionalism adapted from Registered Nurses’ Association of Ontario (RNAO) guidelines (2, 18, 20). The professionalism assessment self-appraisal scale is a 5-point Likert scale to assess their level of professionalism by giving their agreement on a continuum (strongly disagree = 1, disagree = 2, neutral = 3, agree = 4, and strongly agree = 5). To avoid the halo effect, some questions were negatively worded, and the scores of the negatively worded items were reversed; a higher score always corresponds to a more positive value. The third part consisted of 12 questionnaires to measure organizational and professional-related characteristics.

### Data quality control measures

The questionnaires were pretested on 5% (21) of the study participants in Leku Primary Hospital to assess the reliability, clarity, sequence, consistency, and the total time it takes to finish the questionnaire before the actual data collection. The result was not included in the main study. Based on the pretest internal consistency for the self-appraisal scale to measure professionalism, Cronbach’s alpha was 0.948. The data were collected by four BSc nurses supervised by an MSc nurse. Training for data collectors and supervisors was held before the actual data collection period on the objectives of the study and the way of data collection tools. During the training, the following issues are discussed the data collection methods, tools, and how to handle confidentiality of the participants with the data collectors. Each completed questionnaire was evaluated by the principal investigator, and appropriate feedback was sent to the next day. The completeness of the data was handled and stored properly during the data collection.

### Data processing and analysis

All loaded data to Epi-data version 4.6 were exported into the SPSS version 26 software for analysis. The results were described using tables and graphs. Each explanatory variable which has a *p*-value of <0.25 at the bivariate analysis was further fitted into the multivariate logistic regression to control all possible confounding factors. The goodness of fitness was assessed using the Pearson and Deviance goodness test, and there was no multicollinearity among independent variables. The statistical significance of the factors influencing the outcome variable was declared in multivariate logistic regression analysis using an adjusted odds ratio at a 95% confidence interval (CI) at a *p*-value of <0.05.

## Results

### Socio-demographic characteristics of respondents

Among the total number of 413 nurses invited to participate, 405 nurses completed the questionnaire with a response rate of 98%. Out of these, more than half of the participants (55.3%) were females. A larger proportion of the study respondents (42.5%) were found in the age group of 20–29. The majority of the respondents (80.7%) were BSc and MSc holders. Half (50.1%) of the participants were married. In terms of professional working experience, approximately 41% had less than 5 years of work experience. Nearly two-thirds (62.7%) of the participants got their last degree or diploma from governmental institutions (Table 1).

**Table 1:** Socio-demographic characteristics of nurses working in Hawassa city public hospitals, in 2022 (*n* = 405) (2, 21).

Variables	Category	Frequency	Percent
Sex	Male	181	44.7
	Female	224	55.3
Age	20–29	172	42.5
	30–39	161	39.8
	40 and above	72	17.1
Educational status	Diploma	78	19.3
	BSc and above	327	80.7
Marital status	Single	178	44.0
	Married	203	50.1
	Divorced	24	5.9
Working experience	Less than 5	166	41
	6–10	160	39.5
	Above 10	79	19.5
Salary	4,000–6,999	178	31.1
	7,000–9,999	203	58.3
	10,000 and above	43	10.6
Position	Staff nurse	381	94.1
	Head nurse	24	5.9
College of completion	Governmental institution	254	62.7
	Private institution	151	37.3

### Professional related factors

**Figure 2:**
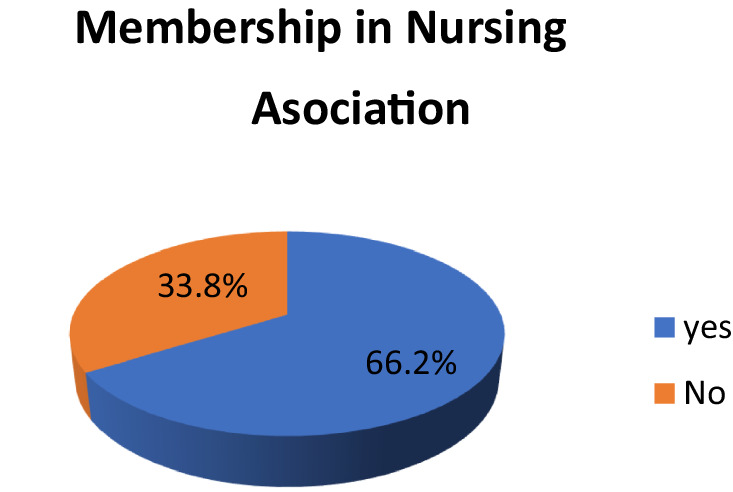
Distribution of nurses in the nursing association.

More than two-thirds (66.2%) of study participants were members of the Ethiopian Nursing Association. Among these, more than two-thirds (68.9%) of the participants had good relations with the health team, and more than one-third (33.8%) of the participants were not members of the Ethiopian nursing association (Figure 2).

### Organization related factors

More than one-third (36%) of nurses claimed that there was good managerial support in the hospital. Nurses who claimed there was an opportunity for education were only 36.3%. Of the study participants, 163 (40.2) claimed that there was workload. Among the participants, 253 (62.5%) reported that necessary instruments are not available in their hospital. Of the total participants, 305 (75.3%) claimed that our hospital does not answer our questions, and 332 (82%) professionals have no life insurance (Table 2).

**Table 2:** Distribution of organization-related factors among nurses working in Hawassa city public hospitals, in 2022 (*n* = 405).

Characteristics	Category	Frequency	Percent
Managerial support	Good	146	36
	Poor	259	64
Instrument availability	Available	152	37.5
	Not available	253	62.5
Administrators are compassionate leaders	Yes	146	36
	No	259	64
The hospital answers questions timely	Yes	100	24.7
	No	305	75.3
Staffs are sufficient to cover the workload	Yes	163	40.2
	No	242	59.8
Opportunity to continue education	Available	147	36.3
	Not available	258	63.7

### Level of professionalism in nursing

**Figure 3:**
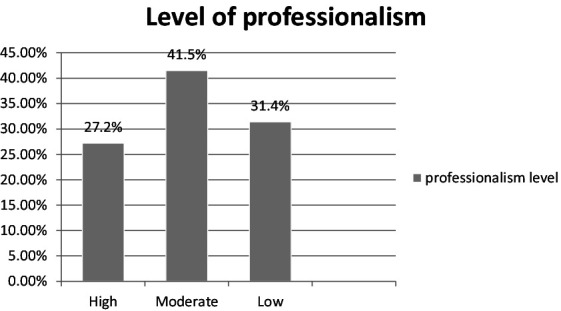
Level of professionalism among nurses working in Hawassa city public hospitals, in 2022 (*n* = 405).

The level of professionalism was found to be on moderate level 168 (41.5%), 127 (31.4%) scored a low level of professionalism, and the remaining 110 (27.2%) scored a high level of professionalism (Figure 3).

Regarding the professionalism scale item, “I have been able to apply my knowledge into practice” and “I have been actively engaged in advancing the quality of care” had maximum score frequency for agreeing, but “I have a body of knowledge that is theoretical and practical” and “I have been committed to life-long learning” had a minimum score frequency for strongly disagreeing (Table 3).

**Table 3:** Description of professionalism scale items among nurses working in Hawassa city public hospitals, in 2022 (*n* = 405).

Professionalism scale items (*α* = 0. 948)	Strongly disagree	Disagree	Neutral	Agree	Strongly agree
	*n* (%)	*n* (%)	*n* (%)	*n* (%)	*n* (%)
I have a body of knowledge that is theoretical, and practical	4 (1.0)	15 (3.7)	95 (23.5)	207 (51.1)	84 (20.7)
I have been actively engaged in advancing the quality of care	5 (1.2)	36 (8.9)	103 (25.4)	209 (51.60)	52 (12.8)
I am engaging in critical thinking about ethical issues in clinical and professional practice	2 (0.5)	50 (12.3)	131 (32.3)	168 (41.5)	54 (13.3)
I am knowledgeable about ethical values, concepts and decision-making	7 (1.7)	37 (9.1)	125 (30.9)	174 (43.0)	62 (15.3)
I have been involved in professional practice initiatives and activities to enhance healthcare	12 (3.0)	52 (12.8)	126 (31.1)	150 (37.0)	65 (16.0)
I have been able to apply my knowledge to practice	8 (2.0)	37 (9.1)	102 (25.2)	189 (46.7)	69 (17.0)
I am recognizing personal capabilities, knowledge base and areas for development	6 (1.5)	33 (8.1)	128 (31.6)	173 (42.7)	65 (16.0)
I am knowledgeable about policies that impact the delivery of healthcare	10 (2.5)	34 (8.4)	130 (32.1)	165 (40.6)	66 (16.3)
I have been committed to life-long learning	4 (1.0)	78 (19.3)	163 (40.2)	129 (31.9)	31 (7.7)

### Factors associated with professionalism

In the final model of multivariate logistic regression analysis level of education, college completion, year of experience, and being a member of professional organizations were found to be significant associations with a moderate level of professionalism among nurses working in Hawassa city public hospitals.

Nurses qualifying for BSc and above were 1.73 times more likely to acquire a higher professionalism level as compared to those who had a diploma (AOR = 1.73; 95% CI: 1.039–2.869). College of completion was positively associated with professionalism in the current study. The odds of being in a higher category of professionalism were 1.54 times higher on average for the nurses who have completed their education in governmental college than private (AOR = 1.54; 95%CI: 1.040–2.272). Being a member of a professional organization was found to have a positive association with professionalism among nurses working in Hawassa city public hospitals, and the odds of being in the higher professionalism category were 1.64 times higher on average for the nurses who were a member of a professional organization as compared to not a member of professional organizations with 61% probability of falling on the higher category of professionalism (AOR = 1.64; 95%CI: 1.090–2.461).

Year of experience was a significant predictor of professionalism among nurses. The odds of being in a higher category of professionalism were 0.44 times lower than on average for those who had less than 5 years of working experience as compared to those who had above 10 years of working experience (AOR = 0.44; 95%CI: 0.247–0.789). Nurses who had more than 10 years of experience had a 55.9% probability of falling into the higher category of professionalism (Table 4).

**Table 4:** Bivariate and multivariate logistic regression analysis of professionalism and associated factors among nurses working in Hawassa city public hospitals, in 2022 (*n* = 405).

		Low	Moderate	High	COR (95%)	AOR (95%)	*p*-value
College of completion	Governmental	66	117	71	0.66 (0.45, 0.96)	1.54 (1.04, 2.27)	0.031*
	Private	61	51	39	1	1	
Educational status	BSc and above	91	141	95	0.76 (0.63, 0.91)	1.73 (1.04, 2.87)	0.035*
	Diploma	36	27	15	1	1	
Year of experience	<5 years	56	82	49	1.75 (1.30, 2.31)	0.44 (0.25, 0.79)	0.006*
	5–10 years	47	64	39	1.30 (0.96, 1.75)	0.63 (0.37, 1.08)	0.092
	Above 10 years	24	22	22	1	1	
Position	Staff nurse	123	159	99	2.40	0.55 (0.25, 1.24)	0.151
	Head nurse	4	9	11	1	1	
Salary	4,000–6,999	49	50	27	1.44 (0.97, 2.13)	0.95 (0.45, 1.98)	0.882
	7,000–9,999	63	108	65	1.10 (0.76, 1.59)	0.97 (0.51, 1.90)	0.973
	10,000 and above	15	10	18	1	1	
Participation in association	Yes	66	122	80	0.66 (0.52, 0.83)	1.64 (1.09, 2.46)	0.018*
	No	61	46	30	1	1	
Gender	Female	75	97	52	1.23 (0.98, 1.52)	0.83 (0.57, 1.21)	0.333
	Male	52	71	58	1		

AOR, adjusted odds ratio; COR, crude odds ratio; 1, reference.Bold values are strongly associated with the dependent variable.

## Discussion

This study aimed to assess the level of professionalism among nurses working in Hawassa city public hospital. The study’s findings showed that 168 nurses (41.5%) had moderate levels of professionalism.

The level of professionalism in the current study among nurses working in Hawassa city public hospitals was found at a moderate level (41.5%). The current finding was consistent with the study done in Iran. However, this study was higher than other studies conducted on Turkish, Japanese, and Jimma (2, 22, 23), which revealed low levels of professionalism. The possible rationale for the discrepancy might be due to the study period and the sample size difference in each study. The other possible rationale could be half of the nurses were members of professional organizations, which gives the nurses different opportunities to update their knowledge through training. As a result, the level of professionalism might be increased (24). Moreover, the educational status of nurses in a developed country was better than developing country; hence, updating qualifications from time to time might increase the professionalism level (25, 26).

On the other hand, the current study finding was low when compared to a study conducted in Arsi and USA which revealed that reported high levels of professionalism (19). The possible reasons for the discrepancy could be low professional development, excessive workload, long working hours, and insufficient services supplied in the nursing profession in developing nations such as Ethiopia (18). For instance in the case of workload, nurse-to-patient ratio in Ethiopia ranges from 1:6 to 1:12 based on the individual institution’s patient load and nurse availability (27). However, the ratio of nurse–to-patient ratio in a developed country such as the USA ranges from 1:1 to 1:6 (28). This could make a difference and affect the nurse’s professionalism level (18).

The other possible reasons could be the negative factors that can hinder professionalism in nursing such as the perception in the community about nursing as a profession, the hierarchic structures of hospitals, the focus of nursing on tasks, a lack of personnel and equipment, low salaries, and weakness in organized labor (22). The healthcare delivery system may also be another explanation for the discrepancy between the study’s findings which can be heavily influenced by the social, technological, and economic development of the countries and this can have an impact on nurses’ professionalism; especially quick changes in the health sector have a pronounced impact on the demand for professional nurses (9).

In the final model of multivariate logistic regression analysis, college completion (governmental), educational level (BSc or above), year of experience (<5 years), and participation in the association were statistically significant with the outcome variable.

Educational level was found to be a strong predictor of nursing professionalism in this study. Nurses with BSc and higher degrees of education had more professional levels compared to diploma holders. This finding was supported by studies done in Turkey and Jimma (2, 22). The possible rationale could be explained by the fact that study participants with a BSc or higher in nursing are more aware of the updated information and difficulties in nursing-related issues that support professional development.

College of completion was another positive predictor of nursing professionals in the current study. The odds of a higher category of professionalism were 1.5 times higher on average for those nurses who have completed their education in governmental college than nurses who were in private. The reason for the problem was rapid and unplanned expansions of private colleges for the sake of opening access, inadequate infrastructure, and limited resources affecting the quality of education (29). The other rationale could be private colleges are on the challenge of balancing poor funding, scarcity of qualified instructors, and poorly qualified students (29, 30). As a result, the knowledge and skill level of nurses were affected. Creating good professional required standards, quality service, and a strong powerful basis in theory, practice, and professional education in the nursing discipline is required to establish professionalism (9).

Nurses who had more than 10 years of experience had a 55.9% probability of falling into the higher category of professionalism as compared with nurses having less than 5 years of experience. This finding was comparable with studies conducted in the USA and Gondar (18, 31). The possible reason could be more experience can lead to expertise in nursing staff, serve as a role model for other nurses, and increase nursing professionalism. This is supported by a study conducted in Saudi Arabia (32). Nurses who were members of professional organizations had 1.64 times higher odds of falling into a higher professionalism category than those who were not. This is supported by the study conducted in the USA (31). This might be because joining professional groups can help with career development, keeping up with the trained issues, increasing skill development, and providing possibilities for professional networking. Moreover, professional associations offer a crucial way to deal with complex challenges that individual members cannot solve and play a key role in assisting nurses in developing their profession (33).

## Conclusion and recommendation

The results of this study indicate that the level of professionalism among nurses at Hawassa Public Hospital was at a moderate level. There was a link between year of experience, part of professional organizations, place of last degree/diploma issued, and BSc or above qualification with the level of professionalism. This suggested that the Regional Health Bureau should collaborate with other responsible bodies to develop various opportunities for nursing staff to increase their professionalism. The minister of health should be focused on private college nurses, nurses lacking the association, and the qualification of the profession. Those hospitals should provide up-to-date training for nurses to increase their level of professionalism. Hawassa University’s comprehensive specialized hospital and respective hospitals should encourage and motivate nurses to participate in professional organizations. Further researchers should conduct research to assess behavioral components of professionalism in nursing in addition to attitudinal components using other designs in multicenter.

### Implication

Educators and managers should implement training to improve the professionalism of nurses, promote patient safety and quality of care, and create an opportunity for nurses to be part of the organization to update their knowledge through training and sharing ideas with senior staff. The minister of health should strengthen nursing professionalism by encouraging professionals issuing their degrees from governmental institutions, to join professional organizations, and provide educational chances more than first degree. Attention should be given to factors that hinder the level of professionalism in nursing.

### Strengths and limitations of the study

The study’s strengths are all governmental hospitals found in Hawassa City were included and an adequate sample was taken from the reference population. However, the study’s drawback is the data were collected through a self-administered question, which may have recall bias. Another limitation of the study is only attitudinal components of professionalism were assessed. Furthermore, because of the study design, we were unable to determine a cause-and-effect relation.

## Data availability statement

The raw data supporting the conclusions of this article will be made available by the authors, without undue reservation.

## Ethics statement

The studies involving humans were approved by The Institutional Review Board (IRB) of Hawassa University College of Medicine and Health Sciences. The studies were conducted in accordance with the local legislation and institutional requirements. The participants provided verbal informed consent prior to completing the self-administered questionnaire which followed guidelines of the Registered Nurses Association of Ontario.

## Author contributions

EA: Conceptualization, Writing – original draft, Writing – review & editing. SM: Supervision, Writing – review & editing. TF: Supervision, Writing – review & editing. MA: Writing – original draft, Writing – review & editing. MT: Writing – review & editing. AT: Writing – original draft, Writing – review & editing.

## References

[1] Vaz De Braganca A Nirmala R. Nurses perception about the public image of a nurse: an exploratory study. An exploratory study (2017).

[2] Solomon Y Beker J Belachew T. Professionalism and its predictors among nurses working in Jimma Zone public hospitals, South West Ethiopia. J Nurs Care. (2015) 5:292. doi: 10.4172/2167-1168.1000292

[3] Merriam-Webster Dictionary. Tolerance. Dari. (2010). http://www.merriam-webster.com/dictionary/tolerance. Diunduh.

[4] Oweis AI. Bringing the professional challenges for nursing in Jordan to light. Int J Nurs Pract. (2005) 11:244–9. doi: 10.1111/j.1440-172X.2005.00536.x, PMID: 16255735

[5] Shamian J El-Jardali F. Healthy workplaces for health workers in Canada: knowledge transfer and uptake in policy and practice. Healthc Pap. (2007) 7:6–25. doi: 10.12927/hcpap.2007.18668, PMID: 17478996

[6] Swisher LL Beckstead JW Bebeau MJ. Factor analysis as a tool for survey analysis using a professional role orientation inventory as an example. Phys Ther. (2004) 84:784–99. doi: 10.1093/ptj/84.9.784, PMID: 15330692

[7] Cruess RL Cruess SR Johnston SE. Professionalism: an ideal to be sustained. Lancet. (2000) 356:156–9. doi: 10.1016/S0140-6736(00)02458-2 10963262

[8] Shohani M Zamanzadeh V. Nurses' attitude towards professionalization and factors influencing it. J Caring Sci. (2017) 6:345–57. doi: 10.15171/jcs.2017.033, PMID: 29302574 PMC5747593

[9] Eid AG Ahmed MZ Safan SM Mohamed SM. Nursing professionalism: a concept analysis. Menoufia Nurs J. (2018) 3:63–9. doi: 10.21608/menj.2018.121319

[10] Azemian A Ebadi A Afshar L. Redefining the concept of professionalism in nursing: an integrative review. Front Nurs. (2021) 8:327–40. doi: 10.2478/fon-2021-0033

[11] Kim YH Jung YS Min J Song EY Ok JH Lim C. Development and validation of a nursing professionalism evaluation model in a career ladder system. PLoS One. (2017) 12:e0186310. doi: 10.1371/journal.pone.0186310, PMID: 29023537 PMC5638508

[12] Wune G Ayalew Y Hailu A Gebretensaye T. Nurses to patients communication and barriers perceived by nurses at Tikur Anbessa Specilized hospital, Addis Ababa, Ethiopia 2018. Int J Africa Nurs Sci. (2020) 12:100197. doi: 10.1016/j.ijans.2020.100197

[13] Ghadirian F Salsali M Cheraghi MA. Nursing professionalism: an evolutionary concept analysis. Iran J Nurs Midwifery Res. (2014) 19:1–10. PMID: 24554953 PMC3917177

[14] Wang L Meric I Huang P Gao Q Gao Y Tran H. One-dimensional electrical contact to a two-dimensional material. Science. (2013) 342:614–7. doi: 10.1126/science.1244358, PMID: 24179223

[15] İşci N Altuntaş S. Effect of professionalism level on tendency to make medical errors in nurses. Florence Nightingale Hemsirelik Dergisi. (2019) 27:241–52. doi: 10.26650/FNJN397503, PMID: 34267978 PMC8127580

[16] Ertem G Oksel E Akbıyık A. A retrospective review about the malpractice applications in medicine. Dirim Tıp Gazetesi. (2009) 84:1–10.

[17] İntepeler ŞS Dursun M. Tibbi Hatalar ve Tibbi Hata Bildirim Sistemleri. Anadolu Hemşirelik ve Sağlık Bilimleri Dergisi. (2012) 15:129–35.

[18] Abate HK Abate AT Tezera ZB Beshah DT Agegnehu CD Getnet MA. The magnitude of perceived professionalism and its associated factors among nurses in public referral hospitals of West Amhara. Ethiopia Nurs Res Rev. (2021) 11:21. doi: 10.2147/NRR.S328749

[19] Tola MD Tafesa F Siraneh Y. Assessment of professionalism in nursing and factors associated among nurses working in Arsi zone public hospitals, Oromia, Ethiopia, 2018. medRxiv. (2020). doi: 10.1101/2020.09.28.20202986

[20] Shen Y Xie W Wang X Qu J Zhou T Li Y. Impact of innovative education on the professionalism of undergraduate nursing students in China. Nurse Educ Today. (2021) 98:104647. doi: 10.1016/j.nedt.2020.104647, PMID: 33189457

[21] Bekalu YE Wudu MA. Level of professionalism and associated factors among nurses working in south Wollo Zone public hospitals, northeastern Ethiopia, 2022. SAGE Open Nurs. (2023) 9:23779608231158976. doi: 10.1177/23779608231158976 36861052 PMC9969456

[22] Dikmen Y Karataş H Arslan GG Ak B. The level of professionalism of nurses working in a hospital in Turkey. J Caring Sci. (2016) 5:95–102. doi: 10.15171/jcs.2016.010, PMID: 27354973 PMC4923842

[23] Tanaka M Taketomi K Yonemitsu Y Kawamoto R. Professional behaviours and factors contributing to nursing professionalism among nurse managers. J Nurs Manag. (2016) 24:12–20. doi: 10.1111/jonm.12264 25355449

[24] Harris M. Benefits of membership in professional organizations. Home Healthcare Now. (2017) 35:129–30. doi: 10.1097/NHH.0000000000000488 28157784

[25] Bryant T Posey L. Evaluating transfer of continuing education to nursing practice. J Cont Educ Nurs. (2019) 50:375–80. doi: 10.3928/00220124-20190717-09, PMID: 31356676

[26] Agyepong EB Okyere ED. Analysis of the concept continuing education in nursing education. J Educ Educ Dev. (2018) 5:96. doi: 10.22555/joeed.v5i1.1598

[27] Weldetsadik AY Gishu T Tekleab AM Asfaw YM Legesse TG Demas T. Quality of nursing care and nurses’ working environment in Ethiopia: nurses’ and physicians’ perception. Int J Africa Nurs Sci. (2019) 10:131–5. doi: 10.1016/j.ijans.2019.03.002

[28] Grondin C Houchens N Gupta A. Quality and safety in the literature: November 2021. BMJ Qual Saf. (2021) 30:921–6. doi: 10.1136/bmjqs-2021-014193, PMID: 34497135

[29] Yirdaw A. Quality of education in private higher institutions in Ethiopia: the role of governance. SAGE Open. (2016) 6:215824401562495. doi: 10.1177/2158244015624950

[30] Melu A. Brief assessment of higher education governance in Ethiopia: reflection on the leap of the decade. J Higher Educ Africa. (2016) 14:107–26.

[31] Kim-Godwin YS Baek HC Wynd CA. Factors influencing professionalism in nursing among Korean American registered nurses. J Prof Nurs. (2010) 26:242–9. doi: 10.1016/j.profnurs.2009.12.007 20637446

[32] Fernández-Feito A Palmeiro-Longo MR Hoyuelos SB García-Díaz V. How work setting and job experience affect professional nurses’ values. Nurs Ethics. (2019) 26:134–47. doi: 10.1177/0969733017700238, PMID: 28393606

[33] Hisar F Karadağ A. Determining the professional behaviour of nurse executives. Int J Nurs Pract. (2010) 16:335–41. doi: 10.1111/j.1440-172X.2010.01849.x 20649664

